# The activation impact of lactobacillus-derived extracellular vesicles on lipopolysaccharide-induced microglial cell

**DOI:** 10.1186/s12866-024-03217-4

**Published:** 2024-02-28

**Authors:** Yanfang Yang, Na Li, Yubo Gao, Fanning Xu, Hui Chen, Chun Zhang, Xinli Ni

**Affiliations:** 1 Department of Anaesthesia and Perioperative Medicine, General Hospital of Ningxia Medical University, Yinchuan, 750004 China; 2 Ningxia Key Laboratory of Cerebrocranial Disease, Ningxia Medical University, Yinchuan, 750004 China

**Keywords:** Probiotics, Lactic acid bacteria, Extracellular vesicles, Microglial polarization, CNS, IL-10, Microbiota-host communication

## Abstract

Perioperative neurocognitive dysfunction (PND) emerges as a common postoperative complication among elderly patients. Currently, the mechanism of PND remains unclear, but there exists a tendency to believe that inflammation plays a significant role in PND. Alterations in the abundance of intestinal microbiota can increase the permeability of the intestinal mucosal barrier and incite extraintestinal inflammatory responses. Metabolites from these microbiota can be absorbed by the intestinal mucosa into the bloodstream, exerting influence upon the central nervous system (CNS). *Lactobacillus* (Lac), serving as an intestinal probiotic bacterium, possesses the capacity to modulate emotional behavior and cognitive functions. *Extracellular vesicles* (EVs) are recognized as novel therapeutic carriers for targeted delivery to regulate physiology and pathogenesis. While the mechanism governing the primary function of Lac-EVs in the CNS remains uncertain. Therefore, we established an in vitro neuroinflammation model to induce PND and then treated the mice with Lac-EVs to observe the effect of these EVs on neuroinflammation, particularly on microglial (MG) polarization. Our research unveils that Lac-EVs reduced inflammation induced by LPS in microglia and the activation of related proteins, including the mRNA expression of M1 labeled protein (iNOS). Moreover, the mRNA expression of M2-labeled protein (Arg1) increased. In addition, flow cytometry revealed that the ratio of M1/M2 microglia also changed significantly. Therefore, Lac-EVs promoted the differentiation of M2 microglia by inducing the preferential expression of specific markers related to M2 macrophages and inflammation. In terms of inflammatory cytokine expression, Lac-EVs decreased the secretion of proinflammatory cytokines (IL-1β and IL-6) and increased IL-10 production after lipopolysaccharide (LPS) stimulation. Therefore, Lac-EVs induce the activation of M2 microglial cells without inducing cellular harm in vitro, and they demonstrate anti-inflammatory effects against lipopolysaccharide-induced neuroinflammation. This finding suggested that it is an effective anti-inflammatory strategy for alleviating inflammation-driven PNDs.

## Introduction

Perioperative neurocognitive disorders (PNDs) are now considered the most common complication among older adults after surgery and anesthesia. Moreover, PND can negatively impact the quality of life for individuals and families, while imposing a significant economic burden on society. PND can diminish the patient’s likelihood of achieving satisfactory functional recovery and raise the post-surgery incidence rate. Consequently, there is an urgent requirement to formulate effective strategies aimed at impeding the advancement of PND and enhancing the quality of life for patients.

The current mechanism of PND is unclear, but inflammation is believed to play an important role in this disease. Surgery and anesthesia can trigger systemic inflammatory responses, and the elevation of proinflammatory mediators (such as chemokines and interleukins) can disrupt the integrity of the blood-brain barrier, resulting in the infiltration of peripheral cytokines into the brain parenchyma. MG cells are intrinsic immune effector components of the CNS, playing a crucial role in maintaining brain homeostasis and responding to neuroinflammation [1]. Neuroinflammatory conditions include MG activation, and MG can assume a number of phenotypes or polarization states in response to different microenvironmental perturbations, with both the M1 proinflammatory phenotype and M2 anti-inflammatory phenotype belonging to two extremes, known as classical and alternative activation, respectively [2]. As macrophages in the central nervous system that play an inherent role in the immune response, MGs are normally in a resting state and always carry out immune surveillance. After MG activation for various reasons, cells can be polarized in two different directions, the M1 (proinflammatory) and M2 (anti-inflammatory) types, and perform different functions by adjusting the local microenvironment. Therefore, adjusting the balance of M1/M2 polarization and preventing neuroinflammatory reactions are highly important for reducing secondary damage to the nervous system. Activated microglia initiate a series of inflammatory events, further activating other microglia and astrocytes. These processes culminate in neuronal injury, functional deficits, and perioperative cognitive impairment. Under conditions of stress and injury, activated microglial cells release inflammatory mediators, resulting in harm to both glial cells and neurons. The M1-type MG polarization can heighten the release of inflammatory agents within the nervous system, hinder neurite growth, encourage PC12 cell apoptosis, and induce circulatory impairment [3]. Conversely, the M2-type MG promotes the resolution of inflammation through anti-inflammatory agents, phagocytosis, the release of trophic factors, elimination of hematoma and debris, and tissue regeneration, thereby deactivating proinflammatory cellular phenotypes and reestablishing homeostasis. Therefore, adjusting the balance of M1/M2 polarization and preventing neuroinflammatory reactions are highly important for reducing secondary damage to the nervous system.

The microbiota-gut-brain-axis (MGBA) reflects two-way, effective communication between the gut microbiota and the CNS [4, 5]. The gut microbiota can influence various pathways, including the immune system, neurochemical signals, bacterial metabolites, and other pathways, to regulate CNS disorders [6]. Recent research has indicated that alterations in the composition of the gut microbiota play a pivotal role in modulating the maturation and function of MG cells in neurological disorders [7]. The changes in the gut flora can impact the permeability of the intestinal mucosal barrier and incite systemic inflammation. Additionally, the gut flora can reduce the expression of tightly linked proteins in the blood-brain barrier; thus, affecting its permeability. This allows the entry of metabolites and secreted chemical signals into the central nervous system, influencing neurons and glial cells, and subsequently impacting brain development and behavior [4, 8]. The gut microbiota serves as a crucial hub within the brain-gut axis, capable of modulating brain development, function, and consequent behavior through this axis. Lactobacillus stands out as one of the primary types of beneficial bacteria in the gut microbiota. Therefore, further investigation is needed to elucidate the precise mechanism of microbial-gut-brain axis in the cross-organ remote regulation of central nervous system processes by Lactobacillus intestinalis.

The important role of probiotics in maintaining human and animal gut health and regulating immune function is increasingly recognized [9]. Numerous researchers employing in vivo and animal model studies have demonstrated that probiotics primarily exert their effects indirectly, such as by modulating the immune system, reinforcing the intestinal epithelial barrier, and competing with pathogens for mucosal adhesion [10, 11]. There is a growing interest in understanding the molecular communication mechanism between probiotics and their hosts. Extracellular vesicles (EVs) are nano-sized membrane-bound vesicles with a phospholipid bilayer structure, secreted by nearly all cells [12]. EVs have been isolated from plants, animals, and microorganisms, with diameters ranging from 20 to 400 nanometers. Microbial-derived EVs contain nucleic acids, lipids, and proteins, enabling them to mediate cell-microbe-host communication. EV-mediated microbe-host interactions can elicit diverse responses and may serve as carriers for the transmission of virulence factors or regulators of inflammatory responses. Currently, EVs have been successfully isolated from various Gram-negative and Gram-positive bacteria [5]. Recent investigations have demonstrated that EVs play a pivotal role in bacterial-bacterial and bacterial-host interactions. Similar to EVs from mammals, EVs derived from probiotic bacteria carry a substantial quantity of proteins, mRNA, and other active factors, facilitating communication between cells, microbes, and the host. The involvement of EVs from probiotics is increasingly recognized compared to previous research [13]. A growing body of evidence indicates that EVs originating from symbiotic bacteria or probiotics also confer benefits in the context of various diseases, engaging with the host through multiple pathways. The initial definition of the beneficial effects of probiotics was centered on living microbial flora for intestinal function. At present, Bifidobacterium and Lactobacillus, as representative probiotics, are the most commonly used bacteria, especially in laboratory research. Research on EVs has focused mostly on their ability to modulate intestinal immune system function or affect intestinal epithelial barrier function [14, 15]. Nonetheless, EVs derived from bacteria have the capability to traverse the blood-brain barrier, and all types of EVs possess the ability to target cells within the CNS [16]. Bifidobacterium species are extensively used as probiotics, and they produce EVs as mediators of the intestinal immune response [17]. *Propionibacterium freudenreichii* generates EVs that harbor proteins known to engage in immunomodulatory interactions with the host [18]. Furthermore, the oral administration of *Lactobacillus paracasei* EVs has been shown to mitigate lipopolysaccharide-induced inflammatory responses in the intestine [19]. Moreover, administering Lactobacillus or other probiotics to mice at the conclusion of surgery and anesthesia can ameliorate anomalies induced by anesthesia/surgery, such as mitochondrial dysfunction, disturbances in the microecological environment, and synaptic loss [20, 21]. In summary, the investigation of the impact of probiotic-derived EVs on CNS cells offers a valuable approach for beneficially modulating CNS disorders. However, many enigmatic questions remain regarding the underlying functions of gram-positive EVs. Most studies conducted thus far have suggested that M1 inhibitors do not yield benefits in the treatment of Alzheimer’s disease (AD). Employing straightforward anti-inflammatory therapeutic approaches may not be effective in the clinical management of neurodegenerative diseases [22]. Merely inhibiting the activation of M1 microglial cells may be inadequate. Simultaneously, the promotion of microglial cells towards the M2 stage may also be imperative for the treatment of neuroinflammatory and neurodegenerative conditions [23]. These findings suggested that an appropriate M1/M2 ratio is critical for neural cell homeostasis. Hence, we postulate that Lac-EVs could serve as a crucial intermediary in the remote control of the M1/M2 microglial ratio by Lactobacillus enterica. Recent investigations have substantiated that EVs originating from bacteria can be absorbed by the gut, traverse the blood-brain barrier, and be taken up by central nervous cells [24]. These findings suggest that Lac-EVs may enter the systemic circulation and regulate MG differentiation across the blood-brain barrier.

In summary, our hypothesis posits that Lac-EVs might facilitate the restoration of cognitive function by modulating the abnormal activation of microglial cells and suppressing neuroinflammation. Consequently, in our research, we established an in vitro experimental model of neuroinflammation to explore the impact of Lac-EVs on the direction of microglial activation induced by LPS.

## Materials and methods

### Bacterial culture and extracellular vesicle extraction

We selected *Lactobacillus* in the current study because many studies have confirmed the beneficial effects of *Lactobacillus* on inflammation [25]. *Lactobacillus rhamnosus* (ATCC 7469) was procured from Biobw Biotechnology Co., Ltd., Beijing, China. It was grown anaerobically in Man Rogosa and Sharpe (MRS) broth media overnight at 37 °C with a stationary state. Of bacterial solution, 200 µL was removed, added to a conical bottle containing 500 mL of MRS, and cultured in a shaker for 18 h. Subsequently, the bacterial solution was centrifuged at 4000×g for 20 min at 4 °C to eliminate impurities, including residual bacteria, cell debris, and polymers. The resulting supernatant underwent filtration using a 0.22 μm Millipore filter [19]. The filtrate was then concentrated and passed through an Amicon®Ultra-15 (100KD membrane) ultrafiltration tube at 4000×g for 6 min at 4 °C. The suspension, which had been concentrated via the ultrafiltration tube, underwent centrifugation using a HIMAC CP70ME (Koki Holdings Co. Ltd CP100NX, Tokyo, Japan) at 150,000×g for 3 h at 4 °C [26]. Subsequently, the supernatant was discarded, and the remaining precipitate was retained as Lac-EVs. The supernatant was subsequently decanted, after which the precipitate was retained as Lac-EVs. The supernatant was removed, and the precipitate was retained as Lac-EVs. The superisolation tube containing the Lac-EVs precipitate was washed three times with PBS. The purified Lac-EVs were stored at − 80 °C.

### Identification of Lac-EVs

#### Nanoparticle tracking analysis for the size and concentration of Lac-EVs

The analysis of the size and particle concentration of the sample was conducted using NanoSight NS300 along with NanoSight NTA software. This process is based on the irregular Brownian motion of nanoparticles within a suspension, influenced by the surrounding solution molecules. By tracking the particle trajectories in the solution, relevant particle size data is acquired. Simultaneously, a high-speed camera and software integrated into the instrument monitor and analyze each observed particle. In this analytical approach, the minimum trajectory length and expected particle size are automatically configured, with a detection threshold set at a minimum of 3 times.

#### Transmission electron microscopy

For the purpose of characterizing the size and morphology of the Lac-EVs, negative staining transmission electron microscopy was employed. The Lac-EVs were dissolved in a solution containing 50 µg/mL PBS. Subsequently, the Lac-EVs were mixed with an equal volume of PBS containing 2% glutaraldehyde and fixed for 5 min. The sample was placed on a copper grid and incubated at room temperature for 20 min. Following this, the copper mesh was immersed in a 5 mL solution of uranyl oxalate at pH 7 for a duration of 5 min, after which it was promptly rinsed with PBS. Subsequently, the sample was placed on a copper grid and incubated at room temperature for 20 min. Finally, the samples were visualized using an H-7650 TEM (Hitachi Ltd., Berkshire, UK) [27].

### Microglial cell culture and Lac-EV treatment

The microglial (MG) cell line BV2 was supplied by Shanghai Zhongqiao Xinzhou Biological Technology Co. Ltd. BV2 cells were maintained in cell culture flasks containing Dulbecco’s modified Eagle’s medium (DMEM) supplemented with 10% fetal bovine serum (FBS, Gibco BRL, Grand Island, NY, USA) and 1% penicillin-streptomycin (PEST, Gibco BRL). These cells were cultivated in a humidified incubator (Thermo Fisher Scientific, Waltham, MA, USA) at 37 °C with 5% CO_2_. The MG cell numbers were determined using a hemocytometer, and the cells were then plated into 6-well, 24-well, and 96-well plastic culture plates as per the specific experimental requirements. The cells were cultured at a density of 1 × 10^4^ cells per well in 96-well plastic culture plates for a CCK-8 assay. Enzyme-linked immunosorbent assays (ELISAs) were performed using 24-well plastic culture plates at a density of 1 × 10^5^ cells per well, after which the cells were cultured to 80% or more confluence.

### Labeling and internalization assay of Lac-EVs

To observe whether Lac-EVs are taken up by microglia, the Lac-EVs were labeled with a lipophilic fluorescent dye (PKH-26; Sigma‒Aldrich, St. Louis, MO, USA) by suspending them in 1 mL of diluent C containing 4 µL of PKH26 fluorescent dye and incubating them at room temperature (5 min). To eliminate excess unbound dye, the sample was treated with 2 mL of 10% BSA at room temperature for 4 min. Subsequently, the mixture was transferred to a 1.5 mL centrifuge tube and subjected to ultracentrifugation at 120,000xg at 4 °C for 70 min. The resulting pellet was resuspended in 100 µL of PBS for further utilization. Approximately 20 µg of labeled Lac-EVs were cocultured with BV2 cells for a duration of 12 h. Following this incubation, the cells were fixed with 4% paraformaldehyde for 10 min and subsequently stained with DAPI. Finally, the uptake of extracellular vesicles was examined using confocal microscopy [28].

### Determination of the optimal concentration of Lac-EVs and BV2 cell coculture by CCK-8

The Cell Proliferation Kit-8 (CCK-8) reagent was used to assess cell proliferation. Microglia were inoculated into a 96-well plastic culture mixture at 100 µL per well. After the BV2 cells were allowed to attach to the wells, an inflammation model was established by adding 1 µg/mL LPS. Then, different concentrations of Lac-EVs (1, 2.5, 5, and 7.5 µg/mL) were added. At 12, 24, and 36 h, the culture medium was removed from the wells, and the wells were gently washed three times with PBS. Following the washes, 100 µL of serum-free high glucose DMEM and CCK-8 solution were added to each well. Following incubation at 37 °C for 1 h, the absorbance at a wavelength of 450 nm was measured using an enzyme marker. In the experiment, both a blank group and a control group were established, each comprising four replicate wells. This procedure was repeated three times.

### Detection of inflammatory factor expression in the coculture supernatant of EVs and BV2 cells by ELISA

Following the instructions, the cocultured supernatant of EVs and BV2 cells was gathered and subjected to analysis using an ELISA kit from Proteintech, Wuhan, China, to assess IL-1β, IL-6, and IL-10 production. In brief, the samples were centrifuged at 1000×g for 5 min at 4 °C, after which the supernatants were collected into 1.5 mL centrifuge tubes. The absorbance of each sample at 450 nm, corresponding to the OD values, was measured using a microplate reader from Rayto Life and Analytical Sciences Co. Ltd, Shenzhen, China.

### RNA extraction and RT‒qPCR analysis

RT-qPCR was performed to evaluate the impact of Lac-EVs on the mRNA expression levels of inflammatory cytokine genes. Total RNA was extracted from cultured cells (in vitro) with TRIzol reagent (R6834–01, Omega). The quantities and purities of the total RNA samples were assessed using ultraviolet spectroscopy (NanoDrop 2000 Spectrophotometer; Waltham, MA, USA), in accordance with the manufacturer’s guidelines. Following the manufacturer’s instructions, RNA (100 *ng*) was reverse-transcribed using the TransScript™ cDNA synthesis kit (Quanshijin, Beijing, China). Subsequently, the transcriptional levels of IL-6, IL-1β, Arg1, iNOS, and IL-10 were determined via RT-qPCR. RT-qPCR reactions were carried out three times for each sample, involving an initial denaturation step at 94 °C for 30 *s*, followed by 40 cycles (94 °C for 5 *s* and 60 °C for 30 *s*). Relative mRNA expression levels were determined by the 2^–ΔΔCt^ method and are expressed as the change in multiples relative to the control group. These data were normalized to the control (GAPDH). The RT‒qPCR primers used are shown in Table 1.

**Table 1:** List of sequences of primers used for RT-qPCR analysis

Gene	Forward primer sequence	Reverse primer sequence
IL-1β	5’-AGTTGACGGACCCCAAAAGATGAAG-3’	5’-GAGTGATACTGCCTGCCTGAAGC-3’
IL-6	5’-AGACTTCCATCCAGTTGCCTTCTTG-3’	5’-TCTGTTGGGAGTGGTATCCTCTGTG-3’
IL-10	5’-CTATGCTGCCTGCTCTTACTGACTG-3’	5’-CTGGGAAGTGGGTGCAGTTATTGTC-3’
Arg1	5’-GAAGACAGCAGAGGAGGTGAAGAG-3’	5’-TGAGTTCCGAAGCAAGCCAAGG-3’
iNOS	5’-GCTTGTCTCTGGGTCCTCTG-3’	5’-CTCACTGGGACAGCACAGAA-3’
GAPDH	5’-AGGAGCGAGACCCCACTAACA-3’	5’-AGGGGGGCTAAGCAGTTGGT-3’

### Flow cytometry analysis

The cellular suspensions of BV2 cells were incubated with anti-CD86 and anti-CD206 fluorescent dye-conjugated antibodies at 4 °C for 30 min (Elabscience Biotechnology Co. Ltd., China) to assess the M1/M2 macrophage balance. The cell suspension was collected and centrifuged at 600 rpm for 5 min. Of bovine serum albumin (BSA), 1 mL was added, and the mixture was centrifuged at 300 rpm for 5 min to wash three times. Of purified anti-mouse CD16/CD32, 2 µL was mixed on ice for 15 min to block nonspecific antibody binding. For cell surface staining, 3 µL of PE-conjugated anti-mouse CD86 (E-AB-F0994B Elabscience) was added to each group, and the samples were incubated on ice for 30 min in the dark, followed by fixation and intracellular staining. After the incubation, 1 mL of staining buffer was added, and the mixture was centrifuged at 300 *rpm* for 5 min. The 200 µL fixed buffer was thoroughly mixed and incubated at room temperature in the absence of light for 30 min. Subsequently, 1 mL of 1x IntraPrep Permeabilization Reagent was added, followed by centrifugation at 600 *rpm* for 5 min. The supernatant was then discarded, and 100 µL of 1x IntraPrep Permeabilization Reagent was added to resuspend the cells. Finally, 3 µL of CD206 (E-AB-F1135D Elabsciennce) was added, thoroughly mixed, and incubated at room temperature for 30 min in the absence of light. It is worth noting that CD206 cells are recognized as M2 macrophages, while CD86 cells are considered M1 macrophages. Flow cytometry analysis was carried out using a FACS Calibur instrument (BD Biosciences, USA) with the assistance of the FlowJo tool. Data from each part were collected using the experimental instrument and processed using FlowJo software 8.1 (FlowJo, Ashland, OR, United States).

### Statistical analysis

The experiments were conducted independently and were replicated at least three times. The data are presented as mean ± standard deviation (SD), with the mean values derived from three to eight measurements for each experiment. Absorbance measurements were standardized using reference conditions. Statistical analysis was performed using GraphPad Prism version 9.0.0 for Windows, GraphPad Software, San Diego, California, USA (www.graphpad.com). One-way ANOVA followed by Dunnett’s multiple comparisons test was employed for certain analyses. Additionally, other statistical tests were carried out using GraphPad Prime 9. Tukey’s multiple comparisons test was used to verify differences between groups. A *p*-value of less than 0.05 was considered statistically significant.

## Results

### Isolation and characterization of Lac-EVs

**Fig. 1:**
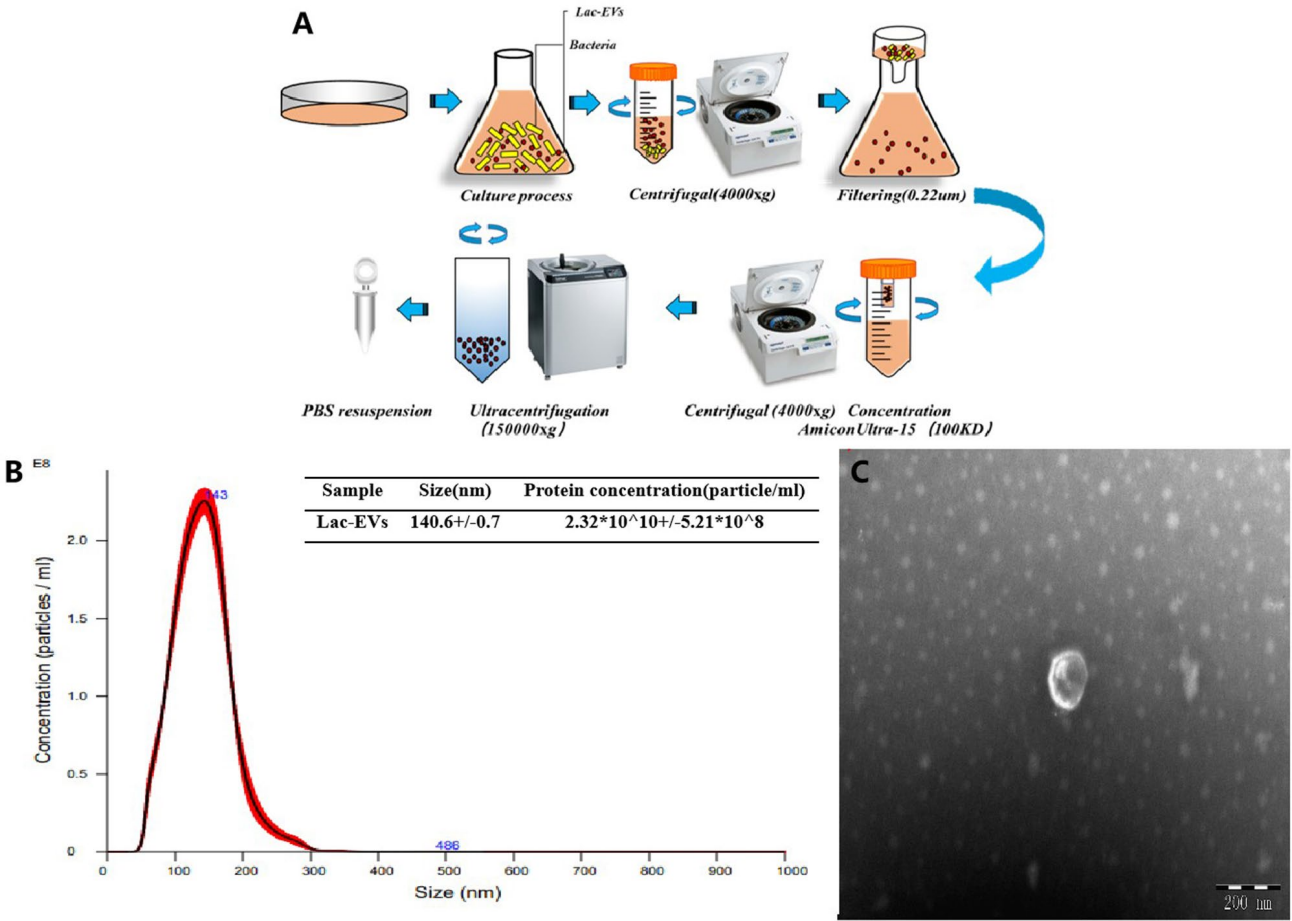
(**A**) Isolation and purification of Lac-EVs by ultracentrifugation. (**B**) Using the NTA to measure the size diameter of Lac-EVs (**C**) The image of transmission electron microscopy of Lac-EVs. *Note* All centrifugation steps were performed at 4 °C. The concentration values are presented as the mean ± standard deviation of at least three values

To confirm the production of extracellular vesicles (EVs) by *Lactobacillus rhamnosus*, we cultivated strain ATCC 7469 in MRS broth at 37 °C for 24 h, and EVs were purified from the cell-free supernatants of stationary phase cultures. The supernatant from the *Lactobacillus rhamnosus* culture underwent purification through ultrafiltration and ultracentrifugation to yield a high-yield of extracellular vesicles. Figure 1A illustrates the extraction process. Nanoparticle tracking analysis (NTA) and transmission electron microscopy (TEM) were employed to characterize the isolated Lac-EVs. Size characterization by NTA showed that the particles of the Lac-EVs were mainly distributed in the range of 70 ~ 400 *nm*, and most of them were approximately 140.6 ± 0.7 *nm* in length (Fig. 1B). Visualization by TEM demonstrated that the Lac-EVs exhibited spherical cup-shaped structures and were composed of lipid bilayers. We found that these EVs were homologous to previously described gram-positive bacterial-derived EVs (Fig. 1C). The above results showed that Lactobacillus species can produce extracellular vesicles. The extracted Lac-EVs were detected with a BCA kit, and the concentration was 1 *µg/mL*; these LEVs were subsequently used in in vitro assays.

### Effects of Lac-EVs on LPS-induced inflammatory responses

**Fig. 2:**
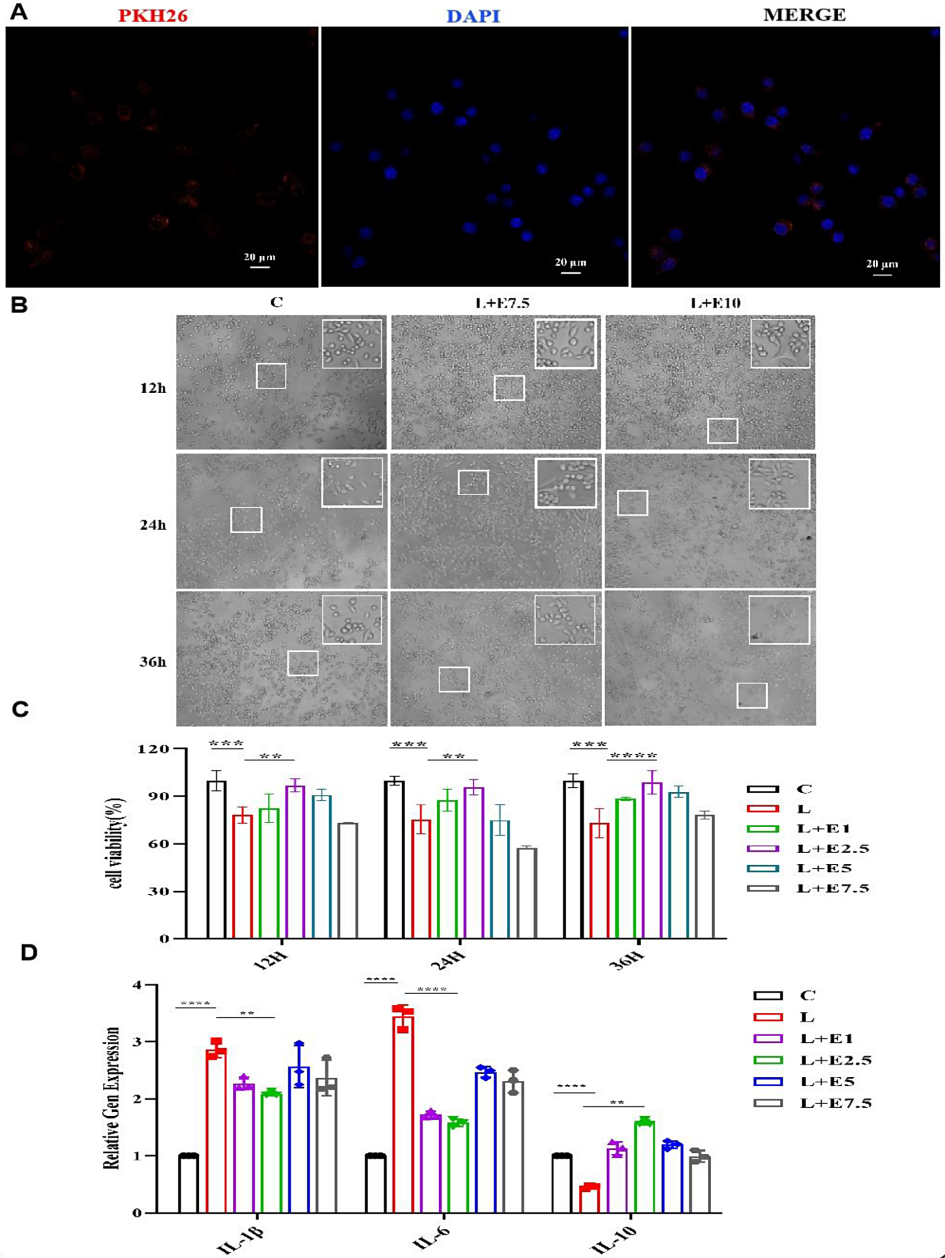
(**A**) Confocal microscopy showing that Lac-EVs were engulfed by MG. The MG cells were coincubated with PKH26-labeled Lac-EVs for 12 h at room temperature. Lac-EVs were labeled with PKH26, and the other material was labeled with DAPI. (**B**) Cell morphology at different concentrations. (**C**) Cell viability of different groups under different concentrations and time points. (**D**) q-PCR analysis of IL-6, IL-10, and IL-1β at a concentration of 2.5 *µg/mL* and after culture for 24 h. The data are expressed as the mean fold change ± SEM of triplicate measurements, and statistical significance was analyzed by one-way ANOVA: *P < 0.05; **P < 0.01; ***P < 0.001; ****P < 0.0001

Bacterial extracellular vesicles were taken up by host cells, facilitating the delivery of biological components to modulate the host immune system [29]. To confirm the validity of our hypothesis, our initial objective was to ascertain whether Lac-EVs could indeed be internalized by BV2 cells, as examined through confocal microscopy. After coculture with microglia for 12 h, we found PKH26-labeled Lac-EVs in the cytoplasm. The results confirmed that Lac-EVs were swiftly engulfed by microglia (Fig. 2A). Several published studies have shown that the concentration of Lac-EVs used for coculture varies within a certain range [14]. To determine the optimal culture concentration of Lac-EVs, we treated BV2 cells with Lac-EVs at different concentrations (20, 15, 12.5, 10, 7.5, 5, and 2.5 *µg/mL*) and cocultured them for 12, 24, and 36 h. The findings indicated that when Lac-EVs were present within the concentration range of 10–20 *µg/mL*, and at the specified time intervals, the cells exhibited amoeboid-like alterations and formed aggregates. The cell bodies enlarged, while the protrusions shortened, and this relationship was found to be dependent on both time and dose (Fig. 2B). Therefore, we cocultured BV2 cells with Lac-EVs at various concentrations (1, 2.5, 5, and 7.5 *µg/mL*) for 12, 24, and 36 h to observe cell viability (Fig. 2C).

We investigated whether Lac-EVs inhibit LPS-induced inflammation through an in vitro cell grouping test. The experimental groups were divided as follows: control group, LPS group, LPS + 1 *µg/mL* Lac-EVs group, LPS + 2.5 *µg/mL* Lac-EVs group, LPS + 5 *µg/mL* Lac-EVs group, and LPS + 7.5 *µg/mL* Lac-EVs group. The optimal concentration of Lac-EVs was determined using a CCK8 assay. As shown in Fig. 2C, in the comparative analysis of the control group, the cell viability of the LPS group decreased. After adding different concentrations of Lac-EVs for treatment, the LPS + 2.*5 µg/mL* Lac-EVs had the greatest effect at different time points. We found that the cell activity in the L + E group was close to that in the control group. The cells were observed under a microscope at a concentration of 2.5 *µg/mL* for 12, 24, or 36 h. The results of the present study showed that the cells cultured for 24 h exhibited the best results. To further verify the rationality of the above results, we evaluated the anti-inflammatory impact of different concentrations of Lac-EVs. We assessed cell viability and the secretion of IL-6, IL-10, and IL-1β, which are known inflammatory cytokines, in MG macrophages treated with Lac-EVs. The secretion of inflammatory factor (IL-6, IL-10, and IL-1β) mRNA was evaluated by q-PCR after 24 h of culture. As shown in Fig. 2D, proinflammatory factor levels decreased, while anti-inflammatory factor levels increased. Therefore, we determined that the optimal concentration of Lac-EVs was 2.5 *µg/mL*, and the cultivation time was 24 h.

### Lac-EVs inhibit the **LPS‑induced polarization of MG toward** the M1 phenotype

**Fig. 3:**
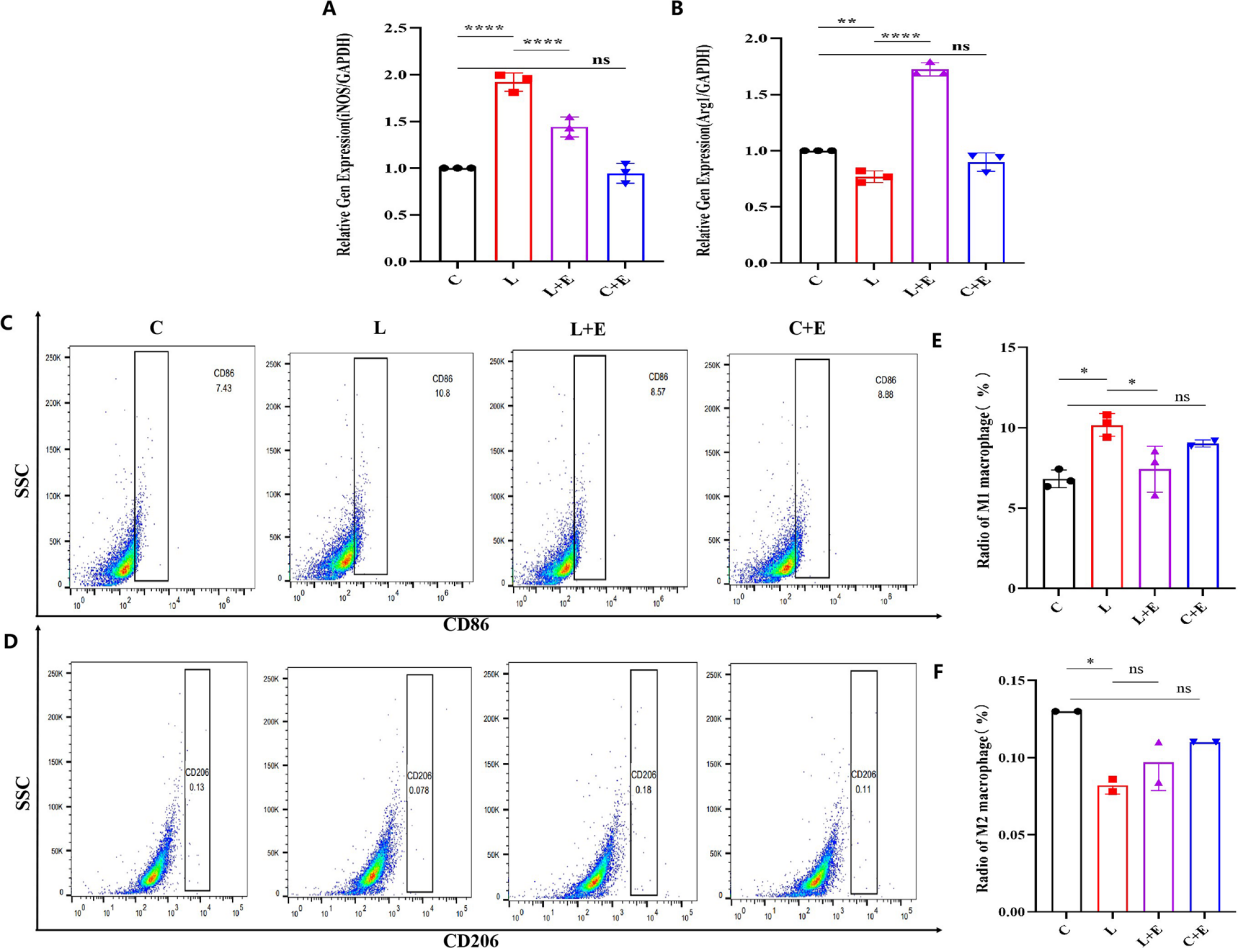
Lac-EVs inhibit M1 macrophage polarization after LPS challenge (**A-B**) The expression of iNOS and Arg1 was measured via RT-qPCR. (**C-D**) Analyses were performed using flow cytometry. The markers of M1 and M2 macrophages were CD86 and CD206, respectively. The ratio of M1 to M2 macrophages is shown in Fig. (**E-F**). The data are shown as the means ± SDs. *P < 0.05; **P < 0.01; ***P < 0.001; ****P < 0.0001)

To observe the effect of Lac-EVs on LPS-induced MG activation, we divided the cells into four groups: the control group (C), lipopolysaccharide group (L), lipopolysaccharide + extracellular vesicle group (L + E), and control group + extracellular vesicle (C + E) group. Lac-EVs strongly induce the differentiation of monocytes into macrophage lines. We performed a thorough analysis of mRNA expression of markers and immune cytokines after Lac-EVs treatment, employing RT-qPCR as previously outlined. This analysis entailed a meticulous evaluation of the expression levels of these markers subsequent to exposure to Lac-EVs. Our objective was to elucidate how Lac-EVs modulate macrophage polarization by comparing the mRNA expression patterns of cells treated with Lac-EVs to those of untreated cells. The results of RT‒qPCR analysis of LPS-treated MGs are shown in Fig. 3. Figure 3A-B shows the mRNA expression levels of M1 (iNOS)-type BV2 cells and M2 (Arg1)-type BV2 cells were measured via RT‒qPCR. The results suggested that the mRNA level of iNOS in the L group was markedly greater than that in the C group, and the expression of this gene was downregulated after the administration of Lac-EVs (Fig. 3A). On the other hand, the mRNA expression level of Arg1 in the L + E group was markedly upregulated compared to that in the L group, while there were no significant differences between the C group and the C + E group (Fig. 3B). These findings indicate that LPS-induced MG leads to an increase in M1 macrophages. In contrast, Lac-EVs treatment reversed the subtle balance of the macrophage phenotype. Therefore, our findings suggest that macrophages treated with Lac-EVs demonstrate a significant inclination toward M2 polarization without inducing cell injury.

Treatment with Lac-EVs altered the balance of proinflammatory M1 macrophages in the presence of LPS, thereby relieving symptoms of CNS inflammation. Additionally, we employed flow cytometry, along with CD86 or CD206 staining, to investigate the infiltration and differentiation of macrophages. The flow cytometry results for CD86 and CD206 showed the same change trend (Fig. 3C-D). We found that the number of activated CD86 macrophages in the LPS group increased and that the number of activated CD86 + macrophages significantly decreased after treatment with EVs derived from Lactobacillus. Similarly, compared with those in the control group, the number of M1 (CD86) cells increased, and the number of M2 (CD206) cells decreased in the LPS group (Fig. 3E-F). When the M1 (CD86) value increases, the M2 (CD206) value decreases, and the overall M1/M2 ratio increases. However, after treatment with Lac-EVs, the tendency of macrophages to differentiate into the M1 phenotype was altered, resulting in a balance of M1/M2 macrophages. In summary, our comprehensive findings demonstrate that Lac-EVs provide significant protection against LPS-induced neurological injury by promoting the polarization of M2 macrophages.

### Lac-EVs **treatment** regulates inflammation-induced expression of macrophage surface markers

**Fig. 4:**
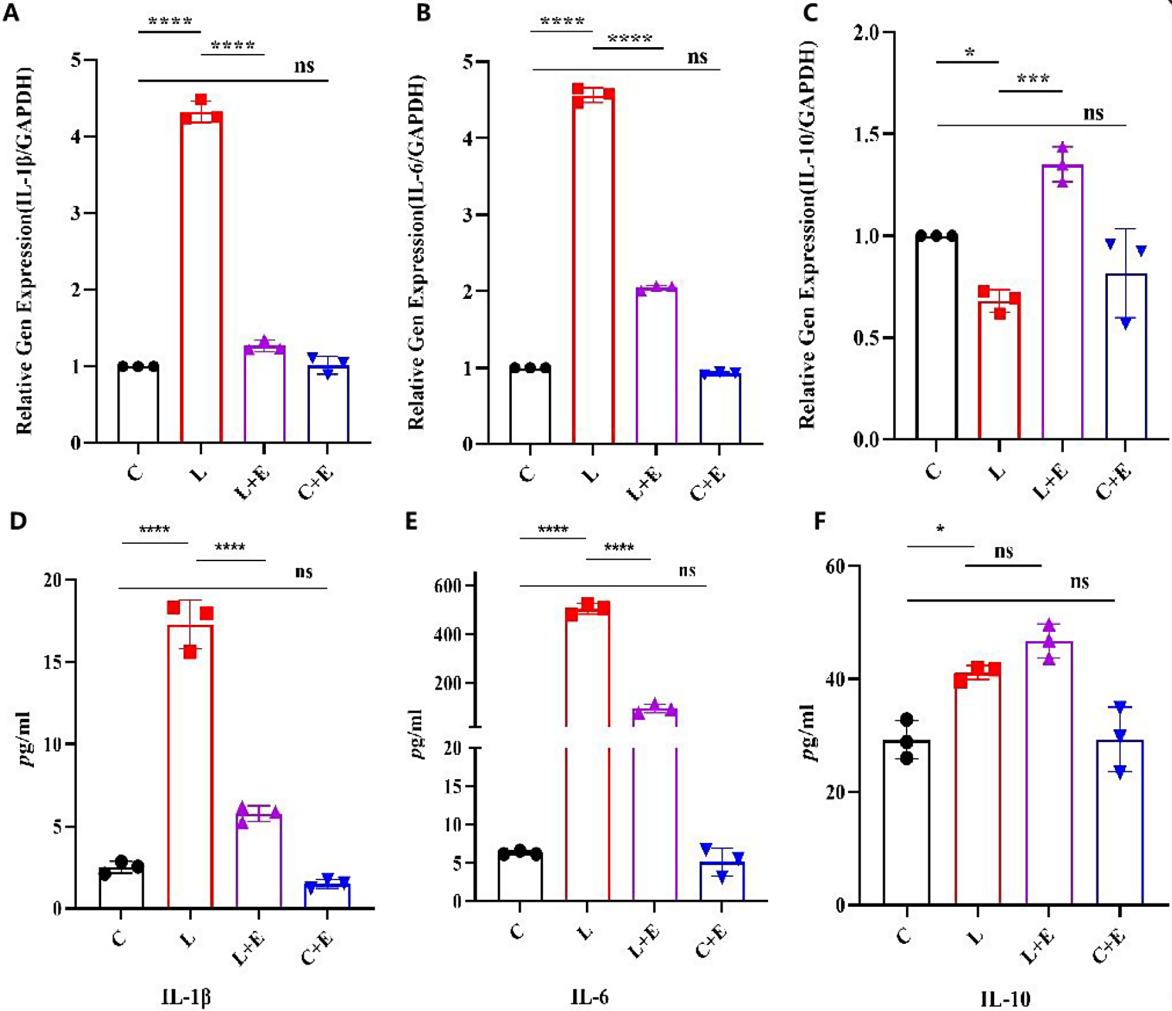
Changes in cytokines in the different groups of MGs were regulated by Lac-EVs. Lac-EVs inhibit the LPS-induced gene expression of IL-1β and IL-6 and the expression of IL-10 under LPS challenge conditions. *P < 0.05; **P < 0.01; ***P < 0.001, ****P < 0.0001; NS, not significant

To gain a deeper insight into the regulatory effects of Lac-EVs on the M1/M2 immune response of MG following LPS stimulation, we explored whether Lac-EVs could mitigate inflammation-related events. LPS was employed to induce inflammatory responses and promote the transformation of monocytes into M1 macrophages. We conducted a quantitative analysis of cytokine production (IL-6, IL-10, and IL-1β) in MG cells utilizing RT-qPCR and ELISA techniques. These findings suggest that the secretion of proinflammatory and anti-inflammatory mediators in the MG is elastically regulated by Lac-EVs (2.5 µg/mL), as shown in Fig. 4. According to the RT‒qPCR results (Fig. 4A‒C), the number of inflammatory factors increased in the LPS-treated group compared with the control group. LPS induced high levels of IL-1β and IL-6 in macrophages from the BV2 group, and the L + E group also exhibited a trend toward decreased expression. However, compared to those in group L, the number of inflammatory factors in group D was lower. Lac-EVs significantly inhibited the secretion of IL-1β (*P* < 0.001) and IL-6 (*P* < 0.05). These findings suggested that Lac-EVs can induce the production of proinflammatory factors but significantly inhibit the expression of proinflammatory mediators induced by LPS, further indicating that Lac-EVs have anti-inflammatory effects (Fig. 4A-B). Importantly, we found that Lac-EVs (2.5 µg/mL) significantly induced the production of IL-10 in LPS-stimulated MG cells, indicating that Lac-EVs may regulate the immune process in MG by adjusting the production of IL-10 (Fig. 4C). According to the ELISA results (Fig. 4D-F), the changes in inflammatory factor levels were similar to those observed via qPCR. These findings indicate that Lac-EVs play a protective role in LPS-induced microglia by regulating the expression of inflammatory mediators. These findings further suggested that Lac-EVs cannot only regulate the M1/M2 ratio to improve the inflammatory response but can also exert immunoregulatory effects by adjusting the secretion of inflammatory factors.

## Discussion

The gut microbiota is indeed a continually changing and dynamic microbial population. It plays an essential role in human digestion, metabolism, immunity, and other processes and is closely connected with the health of the host [30]. Moreover, imbalances exist in the gastrointestinal tract, where imbalances indicate not only various intestinal diseases but also central nervous system diseases. Increasing evidence has revealed the potential link between gut microbial communities and neurodegenerative diseases [31]. Through the regulation of the gut microbial community, it may be possible to prevent and treat neurodegenerative diseases, offering novel approaches for future treatment strategies. In essence, the intestinal microbial community has an impact not only on the host’s metabolic processes but also on brain function and behavior, including cognitive behavior. Disruptions in the ecological balance of the microbial community may play a role in the pathogenesis of PND. Numerous studies have demonstrated the close relationship between immune function and immune homeostasis and the metabolites produced by the intestinal flora [30, 32]. These intricate host-microbiota interactions include the host’s recognition of bacterial metabolites and direct communication with bacteria.

Probiotics are becoming popular functional foods due to their ability to regulate the intestinal flora and inhibit pathogen attacks. Although the concrete protective mechanisms by which probiotics regulate the intestinal flora have still not been fully elucidated, further research and exploration are needed. This includes the intricate process of how probiotics engage with the intestinal microbial communities, exerting specific effects on both the intestinal environment and human health. Consequently, the provision of probiotics is regarded as a promising strategy for restoring microbiota composition and modulating host immune responses. Lactobacillus is a frequently utilized probiotic strain for addressing intestinal disorders. It contributes to the maintenance of gastrointestinal health by regulating the composition of the gut microbiota, preserving the integrity of the gastrointestinal mucosal barrier, enhancing immune function, and employing various other mechanisms. To date, Lactobacillus has been the subject of clinical research in numerous gastrointestinal diseases. However, there is no unified understanding of the function of Lactobacillus. The EVs derived from the microbiota might act as a medium for the delivery of protective components in host–helminth–microbiome crosstalk. Probiotic-derived extracellular vesicles (EVs) have recently garnered significant attention as potential mediators in host-microbiome-probiotic interactions. They possess the capacity to influence the functions of host cells and other microorganisms within the gut microbiota. These EVs play a role in regulating host immune processes, safeguarding the integrity of the gut barrier, and even affecting the composition of the intestinal flora. Consequently, probiotic-derived EVs may offer novel treatment strategies for gastrointestinal disorders [33]. However, further research using animal models and cell experiments is essential to investigate the specific mechanisms and therapeutic effects of probiotic-derived EVs. These research findings indicate that the beneficial effects of certain probiotics are largely reliant on the metabolites they release, such as EVs or the active molecules carried within them. These metabolites possess biological activities that can modulate host cell functions and the composition of the gut microbiota, thereby exerting a positive influence on gastrointestinal health. These compounds have become underlying intermediaries of host immune reactions and anti-inflammatory properties. With the increase in the number of EVs and their potential for treating multiple diseases, EVs have attracted increasing amounts of attention. At present, EVs from different gram-negative and gram-positive bacteria have been successfully isolated [12]. Initially, research on bacterial EVs concentrated on gram-negative and pathogenic bacteria, gram-positive bacterial EVs, and probiotic bacteria has drawn increasing amounts of attention with the improvement of knowledge on health and disease [34]. Numerous studies have confirmed that Lac-EVs have anti-inflammatory effects in colitis models, potentially through modulation of the immune system, protection of the gut barrier, regulation of the gut microbiota composition [33], and stimulation of inflammatory skin conditions [14]. Shen et al. made a significant discovery that EVs produced by commensal bacteria have a notable impact on inflammation [35]. EVs possess the capability to transmit signals to other cells, influencing their function and thereby modulating the inflammatory response. However, research on EVs derived from Lactobacillus has been relatively limited, primarily due to the hindrance posed by the thick cell wall of these bacteria in EV production. In our study, we specifically chose Lactobacillus for EV extraction, and the successful extraction is depicted in Fig. 1. We have not only confirmed the presence of EVs in Lactobacillus rhamnosus (ATCC 7469) but also conducted a comprehensive characterization of their physicochemical, biochemical, and functional properties. The identified EVs exhibit the fundamental characteristics of extracellular prokaryotic membrane vesicles, including a nanometric size range, a cup-shaped morphology, and a spherical structure [18].

Lac-EVs have demonstrated their capacity to mediate immunomodulatory effects in the gut as well as in other biological systems. These effects are likely associated with the release of Lactobacillus secretion mediators; of course, direct interactions between Lactobacillus and immune cells cannot be excluded. Lactobacillus secretion mediators are small molecules or peptides that are secreted by Lactobacillus and have the ability to influence the functions of host cells. Some secretion mediators, such as Toll-like receptors (TLRs), can activate pattern recognition receptors on host cells and trigger downstream signaling pathways that lead to immune responses. Other secretion mediators may engage in direct interactions with immune cells, regulating their functions. Therefore, the release of bacterial secretion mediators by Lactobacillus can contribute to their immunomodulatory effects in the gut [29, 36]. These mediators facilitate the long-distance transport of internal molecules in a concentrated, protected, and targeted manner. They interact with host cells and deliver their cargo to intracellular compartments, thereby influencing host cell functions and modulating host-microbiota interactions. It is important to note that EVs from different bacterial sources may carry distinct cargoes and serve different functions. Further research is warranted to investigate their specific roles in health and disease [37]. To the best of the authors’ knowledge, the immunomodulatory effect of Lac-EVs on experimental neuroinflammation has not been determined. Hence, our hypothesis suggests that Lac-EVs may exert an influence on host immune function through interactions with immune cells. Nevertheless, further research is required to substantiate this hypothesis and to unravel the specific mechanisms involved, as well as to explore potential therapeutic applications. In our experiment, we investigated the effect of Lac-EVs on neuroinflammation-related BV2 cell polarization. Research has shown that Lac-EVs can be effectively internalized by BV2 cells. Canas et al.‘s study of the uptake of EVs derived from L. plantarum by HT29 cells also confirmed this result [38].

The plasticity of macrophages enables them to respond effectively to environmental signals, adapting their phenotype and physiological functions to fulfill specific roles. Macrophages are a vital component of the immune system, with phagocytosis being a fundamental process in which they engulf and digest foreign particles such as bacteria, viruses, and dead cells to eliminate them from the body. Considering that Lac-EVs have the capability to induce monocyte differentiation into the macrophage lineage, which can then be polarized into either M1 or M2 macrophage states, this highlights the potential impact of Lac-EVs on immune responses and macrophage functions. We extensively analyzed the mRNA expression of a series of cell markers related to either M1 or M2 macrophages in response to Lac-EV treatment using RT‒qPCR and flow cytometry. This process is primarily initiated by mimicking the surface characteristics of natural stimuli, such as microorganisms or immune stimulatory molecules, thereby triggering the differentiation and polarization of monocytes into macrophages. Once these cells have differentiated into the macrophage lineage, they can further polarize into either M1 or M2 macrophage states. It is well-established that MG cells maintain an adaptable equilibrium between proinflammatory M1 and anti-inflammatory M2 types, and they can transition from M2 to M1 type in response to tissue injury or infection. As a result, we assessed whether Lac-EVs could help sustain a relative balance in this inflammatory model, potentially promoting the repair and regeneration of injured nervous cells. Macrophages also release various cytokines and other mediators to modulate immune responses and inflammation. Two key enzymes expressed by macrophages during their activation are Arg1 (arginase 1) and iNOS (inducible nitric oxide synthase). Arg1 is typically expressed by macrophages activated by IL-4 or IL-13 and is considered a marker of M2 macrophages, which promote tissue repair and wound healing. In contrast, iNOS is usually expressed by macrophages activated by IFN-gamma and is a marker of M1 macrophages, which have proinflammatory roles in host defense against bacteria and viruses. Inhibition of Arg1 activity can enhance the phagocytic activity of macrophages. While the inhibition of iNOS activity can also enhance phagocytic activity, this aspect is not as thoroughly understood. Inhibition of iNOS activity may result in a shift in macrophage polarization from M1 to M2, which subsequently promotes phagocytosis [39]. Therefore, it is an important versatile modulator in the defense system of macrophages and plays essential roles in many physiological and pathological processes [40]. This research revealed that Arg1 and iNOS activities were markedly changed in BV2 cells treated with Lac-EVs. These findings suggested that Lac-EVs can activate macrophages and achieve specific tasks by altering their surface area.

Immune regulatory cytokines are known to regulate inflammatory responses. Probiotics-EVs have the ability to replicate the anti-inflammatory properties of probiotics by modulating the secretion of immune cytokines. Research studies, particularly those related to acute lung injury, have been conducted in this regard [41]. The findings have shown that LrEVs can help maintain intestinal immune balance and reduce inflammation induced by LPS. In addition, Tong et al. [33] have demonstrated that LGG-EVs exert a certain impact on mitigating colon tissue injury. Another study revealed that LGG-EVs can improve inflammation mainly by inhibiting the activation of the TLR4-NF-κB-NLRP3 axis [42]. In general, LGG-EV treatment effectively suppressed the production of proinflammatory mediators (IL-1β and IL-6). Similarly, Kim et al. [14] This finding also verified this point. Research has shown that LEVs mediate biased expression of cell surface markers and cytokines, resulting in the polarization of THP-1 cells toward the M2 type. Another study carried out in a mouse model revealed that the administration of Lac-EVs effectively reversed the decrease in BDNF and Sirt1 expression induced by GC. As a result, this intervention helped prevent the development of depressive-like behaviors [43]. In another study, a mouse model of colitis showed that EVs excreted from commensal bacteria suppressed the proinflammatory response and accelerated the differentiation of anti-inflammatory macrophages [44]. In in vitro experiments, Choi et al. [45] reported that LpEVs decreased the expression of proinflammatory mediators and cell surface markers induced by LPS and increased the levels of the cytokines IL-10 and TGFβ. In this study, the role of Lac-EVs in altering the population of anti-inflammatory macrophages (M2) in neuroinflammation was explored. Notably, compared with those in the inflammatory group, the Lac-EVs in the Lac-EVs significantly increased the expression of IL-10. The cytokine IL-10 produced by macrophages, plays a pivotal role in preserving the balance between M1 and M2 macrophages. IL-10 not only suppresses inflammation by inhibiting the production of proinflammatory cytokines but also encourages the polarization of M2 macrophages. M1 macrophages primarily function by releasing proinflammatory cytokines, while M2 macrophages exert immunoregulatory effects through the secretion of anti-inflammatory cytokines. Studies have shown that Lactobacillus, as a probiotic, can modulate host immune responses through various mechanisms. In addition to reducing the expression of TNF-α converting enzyme on host cells to dampen inflammation, Lactobacillus can also exert immunoregulatory effects by influencing the activity and function of other immune cells, such as dendritic cells and T cells. These mechanisms contribute to maintaining the balance of the intestinal microecology and assist in the prevention and treatment of immune-related conditions like inflammatory bowel disease [46]. In contrast, M2 macrophages promote cell proliferation and tissue repair through the release of anti-inflammatory cytokines like IL-10. Ornithine and polyamines are involved in promoting macrophage polarization toward the M2 type through the Arg1 pathway. In addition, M2 cytokines (IL-13 and IL-4) promote macrophage polarization toward the M2 type. Reestablishing the balance between M1 and M2 also alleviated inflammation. In the present study, Lac-EVs reconstructed the balance between M1 and M2 during LPS-induced acute inflammation. Macrophages can indeed be polarized in vitro and have the potential for use in cell therapy to treat various diseases. For example, studies have demonstrated that macrophages polarized to the M2 phenotype can facilitate wound healing and tissue regeneration, while those polarized to the M1 phenotype can be utilized to address autoimmune diseases by reducing inflammation and promoting tissue repair. Furthermore, certain small molecule drugs have exhibited the ability to polarize macrophages in vivo, suggesting that macrophage polarization might be a promising target for future drug development. However, more extensive research is required to determine the effectiveness of macrophage polarization as a clinical treatment strategy, potential safety concerns, and any associated side effects. In the context of this study, it is evident that Lac-EVs primarily function as regulators of immune homeostasis. This research underscores the interaction between Lac-EVs and CNS inflammation. By promoting the polarization of M2 macrophages, Lac-EVs alleviate macrophage-related inflammation. Nevertheless, further investigations are warranted to explore the potential application of probiotic-derived EVs in the treatment of neuroinflammation. Future research should concentrate on unraveling the specific mechanisms through which probiotic EVs interact with immune cells and other cell types within the CNS to modulate neuroinflammation. Additionally, studies should evaluate the therapeutic potential of probiotic EVs in animal models of neuroinflammation and assess their safety and efficacy through clinical trials. In sum, the utilization of EVs derived from probiotic bacteria represents a novel and promising approach to addressing neuroinflammation. This emerging field of research may offer fresh insights into the role of EVs in neuroinflammatory disorders and provide innovative therapeutic avenues for managing these conditions. The shortcoming of this article is that it only conducted in vitro experiments and lacked in vivo experiments. In the later stage, we will clarify the effects of Lac-EVs on delirium like behavior, brain tissue damage, inflammatory cytokine secretion, and M1 type MG activation induced by surgical anesthesia at the animal level.

## Conclusions

In our investigation, we delved into the role of Lac-EVs in modulating the two types of macrophages, proinflammatory (M1) and anti-inflammatory (M2), within the context of neuroinflammation. Our research outcomes indicate that Lac-EVs, when administered at optimal concentrations, trigger the release of anti-inflammatory cytokines such as IL-10, CD206, and Arg1 in MG cells. These effects lead to a shift in the proportion of M1-type cells in BV2 cells that have been stimulated by LPS, ultimately polarizing MG cells towards the M2 state in an in vitro setting. Consequently, Lac-EVs can function as anti-inflammatory and immunomodulatory agents, rectifying the imbalance between M1 and M2 macrophages and thereby ameliorating elevated neuroinflammation.

## Data Availability

All data generated or analyzed during this study are included in this published article and its supplementary information files.
